# Correspondence—answer to letter VIAR-D-25–00371

**DOI:** 10.1007/s00428-025-04216-0

**Published:** 2025-08-07

**Authors:** Marit Bernhardt, Benjamin Aretz, Glen Kristiansen

**Affiliations:** 1 Institute of Pathology, University Hospital Bonn, Venusberg-Campus 1, 53127 Bonn, Germany; 2 Institute for Medical Biometry, Informatics and Epidemiology (IMBIE), University Hospital Bonn, Bonn, Germany

We thank Professors Egevad, Delahunt, and Samaratunga for their interest in our study and for their thoughtful critique.

The cited reference, which describes routine full embedding 17 years ago, reflects a pathology landscape that has since evolved [1]. Rising case volumes and workforce constraints have highlighted the need to optimize histopathological workflows—a context in which our study was conducted [2].

We openly reported that in one of 226 cases (0.44%), no tumor was found in the initially selected blocks [3]. This small proportion underscores the importance of thoughtful block selection. As discussed in our manuscript, additional embedding is advisable in cases where underrepresentation is suspected—a practice consistent with real-world diagnostic workflows. Given the rarity of such cases, the incremental effort and time are likely less than that required for universal full embedding. A delay of one or two working days is acceptable in the context of radical prostatectomy processing.

While blinding would be ideal, it was not feasible in this design. We mitigated potential bias through a predefined evaluation protocol, independent assessments by six experienced uropathologists, and consensus reviews in ambiguous cases.

As shown in Table 1, ISUP grade groups were systematically recorded for all radical prostatectomy specimens, and the global ISUP grade group was used for comparison. While selective embedding may miss discrete foci, our sampling protocol aimed to capture representative areas to estimate global grade—a practice consistent with clinical decision-making.

Regarding the “ > 5% change” threshold, we clarify that this refers to relative shifts in the proportion of any Gleason pattern [3, 4, or 5], not cribriform growth. Cribriform pattern was recorded categorically and demonstrated high concordance between embedding strategies, with statistically confirmed non-inferiority.

Tumor volume was not assessed due to known limitations in accuracy and ongoing debates about its independent prognostic significance. Nonetheless, the average prostate weight (41.1 g) and the proportion of pT3 tumors (43.4%) are in line with typical tertiary center cohorts. Contrary to the critique, statistical testing for non-inferiority offers more nuanced insights than classical nonparametric methods [4]. A subgroup analysis in pT2 tumors confirmed non-inferiority for R status and presence of IDC-P; cribriform growth narrowly failed, likely due to reduced statistical power. A larger, prospective study is currently underway to address this aspect more robustly.

Importantly, our conclusions apply specifically to the defined selective embedding protocol and its performance regarding selected outcome measures. We do not advocate replacing full embedding universally but propose standardized selective embedding as a validated alternative—particularly relevant in high-throughput or resource-limited settings. Finally, we would like to emphasize that even so-called full embedding entails only a segmental evaluation of far less than 1% of the prostate, inherently constrained by the slicing intervals [4]. Our protocol simply broadens these intervals while maintaining diagnostic representativeness. We appreciate the authors’ engagement and hope this response clarifies our study’s aims, methods, and limitations.
